# Macrophage inclusions in cerebrospinal fluid following treatment initiation with antisense oligonucleotide therapies in motor neuron diseases

**DOI:** 10.1186/s42466-023-00305-0

**Published:** 2024-02-22

**Authors:** Maximilian Vidovic, Mario Menschikowski, Maren Freigang, Hanna Sophie Lapp, René Günther

**Affiliations:** 1 Department of Neurology, University Hospital Carl Gustav Carus, Technische Universität Dresden, Fetscherstraße 74, 01307 Dresden, Germany; 2 Institute of Clinical Chemistry and Laboratory Medicine, University Hospital Carl Gustav Carus, Technische Universität Dresden, Fetscherstraße 74, 01307 Dresden, Germany; 3 German Center for Neurodegenerative Diseases (DZNE) Dresden, Tatzberg 41, 01307 Dresden, Germany

**Keywords:** ALS, SOD1, SMA, Antisense oligonucleotide therapy, Nusinersen, Tofersen, Macrophages, Macrophage inclusions, Asophages

## Abstract

5q-associated spinal muscular atrophy (SMA) and amyotrophic lateral sclerosis (ALS) are two distinct neurological disorders leading to degeneration of lower motor neurons. The antisense oligonucleotides (ASOs) nusinersen and tofersen are novel disease-modifying agents for these diseases, respectively. In the context of ASO treatment, the cytological characteristics and composition of cerebrospinal fluid (CSF) have recently garnered particular interest. This report presents a case series of CSF cytology findings in two patients with SMA and ALS revealing comparable unspecified macrophage inclusions following treatment initiation with nusinersen and tofersen. Yet, the presence of these “asophages” in the treatment course of two different ASOs is of unclear significance. While both treatments have been well tolerated, this phenomenon warrants attention, given the long-term nature of these treatments.

## Introduction

5q-associated spinal muscular atrophy (SMA) and amyotrophic lateral sclerosis (ALS) are aetiologically different motor neuron diseases sharing the degeneration of lower motor neurons. The antisense oligonucleotide (ASO) nusinersen was the first approved gene therapy for patients with SMA increasing the production of functional survival motor neuron (SMN) protein [1, 2]. The ASO tofersen was developed for patients with *superoxide dismutase 1 (SOD1) mutation*-associated ALS (*SOD1-*ALS) to reduce the toxic SOD1 protein level [3, 4]. The U.S. Food and Drug Administration (FDA) recently granted accelerated approval for tofersen, that is currently being further evaluated in the VALOR open-label extension phase and available for patients with *SOD1*-ALS in early access programs in Germany and other countries outside the USA.

Unclear inclusion bodies in macrophages following nusinersen treatment initiation were described in a few recent studies [5–7]. So far, only one case report has been published, describing similar inclusions in macrophages of patients with *SOD1*-ALS following treatment initiation with tofersen [8]. Here, we present two cases: one involving a patient with SMA treated with nusinersen, and the other featuring a patient with *SOD1*-ALS treated with tofersen. Comparing both cases, similar inclusions in macrophages within the cerebrospinal fluid (CSF) emerged following the initiation of ASO treatment.

## Case Report

### Patient 1 – SMA

**Fig. 1:**
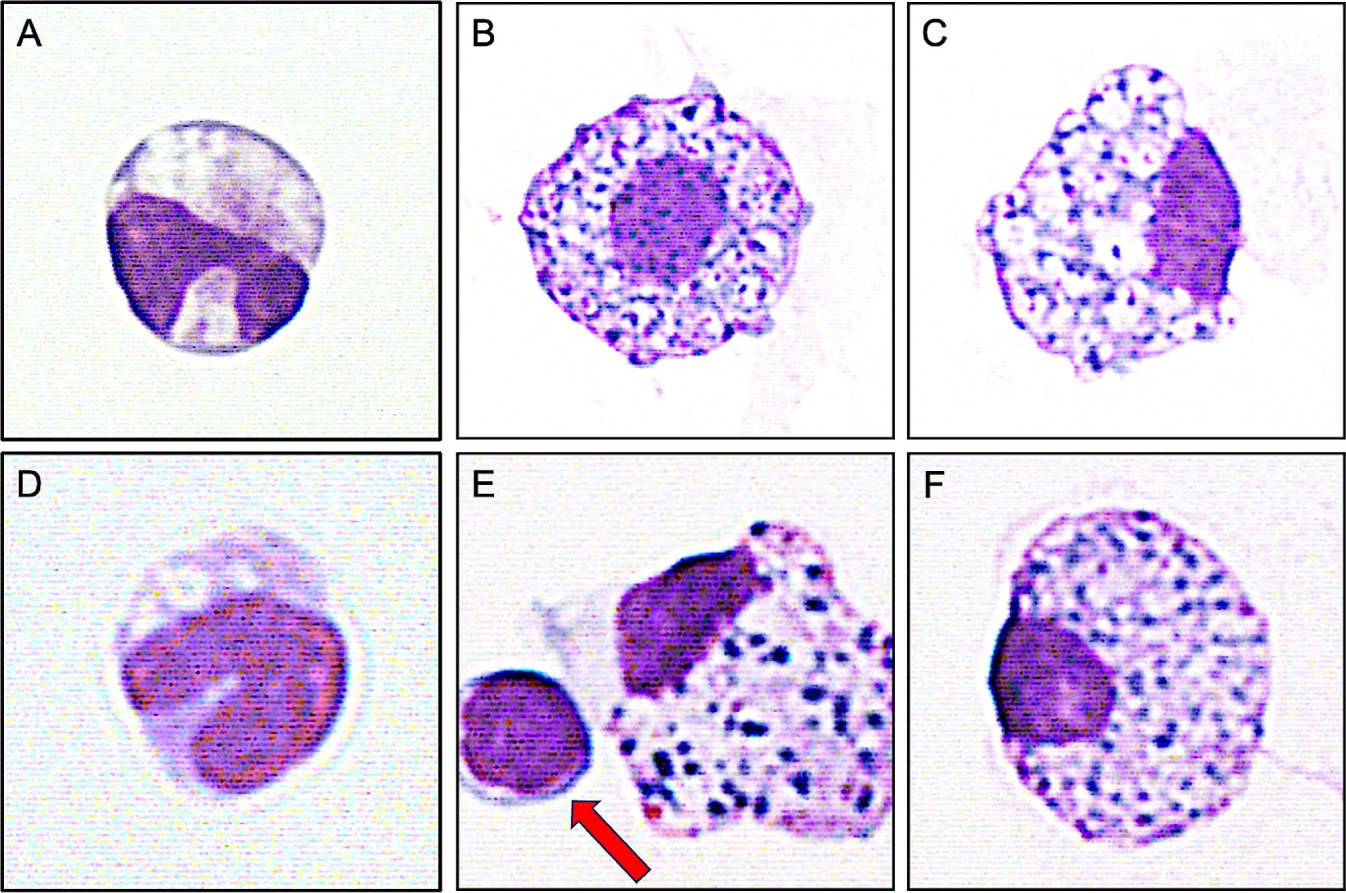
Macrophages of an adult patient with SMA (**A**) and an adult patient with *SOD1*-ALS (**D**) before ASO treatment (treatment-naive): Normal configuration with typical irregular nuclear contour and multiple intracytoplasmic vacuoles (1000x, May-Grunwald-Giemsa staining). Macrophages of the same adult patients with SMA (**B** & **C**) and *SOD1*-ALS (**E** & **F**) during ASO treatment presenting with eosinophilic and basophilic inclusion bodies. Occasionally, macrophages with inclusions appeared with contact to lymphocytes (**E**, red arrow) (1000x, May-Grunwald-Giemsa staining). ASO: antisense oligonucleotide; SMA: 5q-associated spinal muscular atrophy; *SOD1-*ALS: *superoxide dismutase 1 mutation*-associated amyotrophic lateral sclerosis

We investigated a 61-year-old female patient with SMA type 3. She was diagnosed with SMA at the age of 15 and has received 17 injections of nusinersen within 54 months since April 20, 2018. Nusinersen was administered according to the recommended dosing schedules with four loading doses on treatment days 0, 14, 28 and 63, followed by maintenance doses every 4 months. Each application contains 12 mg nusinersen and was injected intrathecally via lumbar puncture after obtaining CSF. During the treatment period, no clinically significant adverse events have been observed yet. The patient did not suffer from significant scoliosis and had no history of spinal surgery. Before nusinersen treatment, CSF cytology findings showed macrophages with normal configuration (Fig. 1A). The patient presented a CSF with normal leucocyte cell count, normal total protein and albumin concentrations and without intrathecal synthesis of immunoglobulins (Igs). Unclear inclusions within macrophages were first observed during the loading phase on treatment day 63 (Fig. 1B & C). Subsequently, these inclusions were detected on treatment months 14, 26, and 30, and consistently since treatment month 38. No relevant changes were observed with regard to leucocyte cell count, total protein and albumin concentration and presence of Igs in CSF during treatment course (Table 1).

**Table 1:** Cerebrospinal fluid findings following treatment initiation with nusinersen and tofersen. T1: treatment-naive; T2: month 6 of nusinersen treatment/month 2 of tofersen treatment (after completed loading phases); T3: month 54 of nusinersen treatment/month 9 of tofersen treatment

		Patient 1 – SMA:			Patient 2 – *SOD1*-ALS:		
	Reference values	T1	T2	T3	T1	T2	T3
Leucocyte count (MPt/L)	< 5	3	4	1	1	3	21
Protein (mg/L)	150–450	249	262	214	483	764	1235
Albumin (mg/L)	< 350	129	137	134	268	558	879
Albumin ratio (x10^− 3^)	(4 + Age/15)	3.37	3.58	3.41	5.60	12.85	18.65
IgG (mg/L) IgG synthesis (%)	10–30 negative	14.1 0.0	12.8 0.0	12.53 0.0	44.48 1.5	69.09 0.0	115.63 0.0
IgA (mg/L) IgA synthesis (%)	1–3 negative	1.29 0.0	1.58 0.0	1.74 0.0	5.81 0.0	10.78 0.0	17.94 0.0
IgM (mg/L) IgM synthesis (%)	0.5–1.5 negative	< 0.5 0.0	< 0.5 0.0	< 0.12 0.0	0.33 0.0	1.82 0.0	12.26 62.6
OCBs (CSF/Serum)	negative	Type 4	Type 4	Type 1	Type 4	Type 1	Type 4
Lymphocytes (%)	50–90	93	88	97	94	85	85
Monocytes (%) Macrophages with unspecified inclusions	10–50 none	6 –	9 +	1 +	3 –	9 +	10 +

**Ig: immunoglobulin; SMA: 5q-associated spinal muscular atrophy;*SOD1*-ALS**: ***superoxide dismutase 1 mutation*-associated amyotrophic lateral sclerosis; OCBs: oligoclonal bands; Type 1: no evidence for intrathecally produced OCBs; Type 4: equal numbers of matched OCBs in CSF and serum**

### Patient 2 – *SOD1*-ALS

This patient was a 39-year-old female diagnosed with *SOD1*-ALS (c.358-10T > G) in February 2022. She has received 11 injections of tofersen within 9 months since October 22, 2022. Tofersen was administered according to the recommended dosing schedules with three loading doses on treatment days 0, 14 and 28, followed by maintenance doses every month. Each application contains 100 mg tofersen and was injected intrathecally via lumbar puncture after obtaining CSF. Except for a pseudoradicular pain event after the first injection, no further clinically significant adverse events have occurred. Before tofersen treatment, CSF cytology findings revealed normal macrophages without inclusions (Fig. 1D), normal leucocyte cell count and normal albumin concentration. Only CSF total protein as well as IgG concentration was slightly increased. Following treatment initiation, macrophage inclusions were first observed on treatment day 28 (Fig. 1E & F). There was an observation of mild pleocytosis, along with elevated total protein and albumin concentrations since treatment month 3. Simultaneously, intrathecal immunoglobulin M antibody (IgM) synthesis was detected since treatment month 5, steadily increasing by treatment month 9. Equal numbers of matched oligoclonal bands (OCBs) in CSF and serum (Type 4) were evident before loading phase and on treatment month 9 (Table 1).

## Discussion

This case series reports non-specific inclusions in macrophages in two patients diagnosed with SMA and *SOD1*-ALS. Both motor neuron diseases are characterized by distinct pathomechanisms and ASO treatment approaches: SMA arises from loss-of-function mutations in the *SMN1* gene. Nusinersen improves the integration of exon 7 into the mRNA, thereby augmenting full-length SMN protein levels [2]. Conversely, *SOD1*-ALS primarily results from a toxic gain-of-function mutation in the *SOD1* gene. Tofersen targets mRNA produced by mutated *SOD1* genes, effectively diminishing the synthesis of toxic SOD1 proteins [3].

In both patients, similar unspecified numerous eosinophilic and basophilic inclusion bodies were observed inside intracytoplasmic vacuoles of macrophages following treatment initiation with nusinersen and tofersen. For this common cytological phenomenon of new unspecified macrophage inclusions after treatment initiation of different ASOs, we suggest to use the term “asophages”. The composition of these inclusions is still unknown, however it is rather unlikely that they are comprised of ASO particles [7]. One hypothesis would be that these inclusions reflect activation of the innate immune system. In line with this, we observed increased concentrations of the monocyte activity marker chitotriosidase 1 in CSF of patients with SMA during nusinersen treatment [9]. Occasionally, lymphocytes were found in close proximity to these macrophages suggesting an antigen-presenting interaction.

Both patients exhibited distinct patterns in terms of cellular and humoral components in the CSF. Tofersen treatment was associated with mild pleocytosis, as well as increased protein and albumin concentration. These findings align with recent studies, though the underlying cause remains unclear [3]. Additionally, we observed elevated Ig levels and intrathecal IgM synthesis, indicating a potential activation of the humoral immune system. Nusinersen treatment was not associated with intrathecal Ig synthesis. However, mild changes in CSF routine parameters were also confirmed in adult patients with SMA under nusinersen treatment [10].

The prevalence of “asophages” remains speculative due to a lack of comprehensive systematic cohort studies. However, in a recent small cohort study of adult patients with SMA, macrophage inclusions were observed in all patients (n = 19) [5]. To our knowledge, no similar studies of patients with *SOD1*-ALS are published yet.

To date, nusinersen and tofersen were shown to be well tolerated and none of our patients experienced significant adverse events. “Asophages” appear to exhibit a consistent pattern in patients undergoing various ASO treatments, persisting even in later stages of the treatment courses. However, their composition and functional role are unknown and need to be investigated in subsequent studies, eventually contributing to a more comprehensive understanding of the benefit-risk ratio associated with ASO treatments.

## Data Availability

Data and material that support the findings of this study are available from the corresponding author upon reasonable request.

## Abbreviations

ALS: Amyotrophic lateral sclerosis
ASO: Antisense oligonucleotide
CSF: Cerebrospinal fluid
IgM: Immunoglobulin M antibody
Igs: Immunoglobulins
OCBs: Oligoclonal bands
SMA: 5q-associated spinal muscular atrophy
SMN: Survival motor neuron
SOD1: Superoxide dismutase 1
*SOD1*-ALS: *Superoxide dismutase 1*-associated amyotrophic lateral sclerosis
